# Childhood Caries Management

**DOI:** 10.3390/ijerph19148527

**Published:** 2022-07-12

**Authors:** Hai Ming Wong

**Affiliations:** Paediatric Dentistry and Orthodontics, Faculty of Dentistry, The University of Hong Kong, 34 Hospital Road, Hong Kong SAR, China; wonghmg@hku.hk

## 1. Introduction

Dental caries, also known as tooth decay or cavities, result from the breakdown of teeth due to bacterial acids. The term caries was first coined in the early 17th century. In Latin, caries means either “rottenness” or “decay”, which is an appropriately apt description of this disease. Further definition or terminology of dental caries specificity also exists. For example, the currently accepted term of “early childhood caries” (ECC) is defined as the presence of at least one carious lesion on a primary tooth in a child under the age of 6 years old [1]. ECC was previously known under such aliases as “bottle caries”, “baby bottle syndrome”, “baby bottle tooth decay”, “nursing bottle caries”, “nursing caries”, or “prolonged nursing habit caries”. The origins of these caries nomenclatures are derived from past observations that tooth decay was frequently associated with particular events such as children falling asleep attached to bottles containing sweetened liquids. “Rampant caries” is another pattern of tooth decay, signifying advanced or severe decay amongst multiple surfaces of numerous teeth. Rampant caries is frequently seen in children with poor oral hygiene, high sugar intake based on poor macronutrient diets and sweetened medication, radiation to the head and neck, and others.

It is important to recognize that the presentation of caries is highly variable. Caries is initially clinically diagnosed as the first presence of a small chalky area within a smooth surface. If left untreated, complications from uncontrolled initial caries may gradually worsen and its presence becomes larger, cavitated, and symptomatic. Symptoms from cavities include discomfort, sensitivity and pain; later complications include inflammation of the pulp tissue or tissue surrounding the specific tooth, acute and chronic infection, abscess formation, tooth loss, and cellulites. Given its large public health importance and awareness, the call for manuscripts for the *International Journal of Environmental Research and Public Health* Special Issue “Childhood Caries Management” was proposed at the beginning of 2022 as part of multidisciplinary efforts to address this important topic. The current state of knowledge and conventional and innovative strategies for the management of childhood caries are summarized in the following sections.

## 2. Prevalence, Burden and Impact of Childhood Caries

Currently, the most common health condition in the world is dental caries. There are more recent advancements and implementations of dental caries management, which has helped alleviate its burden in certain contexts, but not in all. For example, there has been a gradual decline in the prevalence of dental caries in children of all ages in western countries, but caries, specifically in preschool children, remain a major public health problem in both developed and developing countries. The prevalence of caries also varies widely depending on several factors such as ethnicity, culture, socioeconomic status, lifestyle, dietary patterns, oral hygiene practices, and inherent genetic-based developmental defects in the enamel [2,3]. The prevalence rate of ECC in most developed countries is less than 15%. In contrast, the prevalence can be as high as 50–80% in less developed countries, the wide range considered a reflection amongst varying subsets of disadvantaged groups within these countries [4,5]. The high prevalence of caries in some contexts may be partly associated with the intergenerational effect of the relatively low education attainment of caretakers [6].

In examining the scope of caries burden worldwide, young children demonstrate, by far, the highest caries burden across all age groups. In 2017 alone, the prevalence of caries cases in deciduous teeth was over half a billion worldwide, representing an age-standardized prevalence rate of 46.9% and 39.3% cases among 1–4 and 5–9 year-olds, respectively. Caries disproportionally affects vulnerable children who are socioeconomically disadvantaged. The statistics are clear; in 2019, 84.5 million incident cases of caries globally were attributable to sociodemographic inequality. Translating these numbers into real-world contexts means an average of 2.7 new caries cases every second, and these cases are directly attributable to inequality. The effect of inequality on caries cases becomes further exacerbated based upon the degree of inequality. In fact, it is found in a study on the relationship between caries burden and inequality that a comparably high degree of inequality was observed across all countries within the poorest quintile over the last three decades [7].

The symptoms of caries are not the only factors that can alter the child’s quality of life. Indirect consequences of caries need to be accounted for, such as altered eating and sleeping patterns, risk and anxiety of emergency visits and hospitalization, and the loss of school attendance, which can result in loss of invaluable learning time. Given the socioeconomic difficulties affecting the majority of caries cases, the latter consequence is not insignificant as the lack of resources makes the loss of time in the classroom irreplaceable to growth and development. In particular, there are many health consequences of ECC in small children. In the majority of very small children, ECC is significantly associated with both physical growth and weight gain. ECC causes tooth pain severe enough to result in insufficient food intake that is unable to meet metabolic growth. This development disproportionally affects children less than 2 years old, although it can and does affect children in all growth stages. Pain and subsequently stress caused by ECC also result in overactive glucocorticoid production which greatly impacts overall health and specifically leads to disrupted sleep duration and quality. In addition, ECC leads to depressed erythrocyte production and consequently the suppression of hemoglobin [8]. High severity of ECC resulting in dental decay and subsequently early tooth loss have been associated with impaired speech development, malocclusion, and reduced self-esteem [9]. ECC also has significant consequences on family dynamics. Research has showed an association between ECC and child maltreatment, as exemplified by emotional outbursts and the threat of or actual violence [10]. Health consequences from ECC not only occur in the short term but have far lasting complications into the future. Children with their first experiences in caries as infants or toddlers have a greater probability of subsequent caries in both the primary and permanent dentitions. In cases with severe caries, children are likely to continue having oral and overall general health issues for which treatment costs are often financially out of reach for their parents [11]. Research has shown that caries in deciduous teeth are predictors of oral health in later life. ECC predisposes these children to future noncommunicable diseases in adulthood, and also has profound negative impacts on families and societies independent of the individual. Given the exponential negative health consequences that ECC bears, future oral health policies focused on child intervention strategies should be expedited given the current stalled improvements in the control of caries development. In addition, oral health promotion programs should also target parents and caregivers essential in sustaining the reduction in the caries burden, given their central roles in child development. These programs should be introduced particularly in low-resourced environments and countries given the inequality gaps present within predisposed, vulnerable parents residing in these regions.

Child health professionals, including but not limited to physicians, physician assistants, and nurses, can play a significant role in reducing the burden of caries prevalence. Although the majority of children do not visit a dentist until the age of 3 years old, children have visited other health professionals up to 11 times for by this age. For a myriad of reasons, whether lack of funding, poor public health awareness, or limited widespread attention based on higher perceived importance on other health issues, amongst others, ECC intervention remains relatively poor. Thus, for the most part, ECC remains untreated despite the high prevalence of caries among young children.

Furthermore, the presence of untreated carious teeth is a significant predictor of poor oral health-related quality of life [4,9]. The financial burden from dental treatment is particularly pronounced in lower socioeconomic groups; thus, many children clinically diagnosed with caries will not receive adequate treatment based solely on financial hardships. Those fortunate enough to afford adequate dental health care are not necessarily guaranteed a good bill of health. Dental treatments itself are hindered greatly by children’s fear of the dental drill or anesthetic injection [12], and/or lack of co-operative ability during treatment based on young age or specific disabilities [13]. One also has to factor in the most recent events as reasons for poor oral health outcomes in children. The sobering inequality of the caries burden has been exacerbated during the COVID-19 pandemic. COVID-19 has resulted in severe disruptions to school attendance as well as the temporary suspension of impactful school-based oral health programs [14]. Given the multifactorial dynamics of ECC, the management of childhood caries requires prevention at the individual and population levels to address all the root causes of ECC, such as socioeconomics, family dynamics, and inter-individual fear.

## 3. Prevention, Diagnosis and Treatment for Childhood Caries

Oral hygiene maintenance and dietary modification are the two primary home-care methods for caries prevention. Yet the current caries burden strongly suggests that these daily tasks remain too difficult for many children to put into any type of routine practice. Aside from home-care methods, professional dentistry methods include fluoride application on smooth tooth surfaces and sealant application to pits/fissures in response to tooth eruption are the most common strategies for caries prevention with proven efficacy in the clinical setting. Special applications such as xylitol, amorphous calcium phosphate, silver diamine fluoride, systemic fluoride, or behavioral-based interventions such as oral health assessment, motivational interviewing, social story, and theory-based oral health intervention are also recommended by dentists as alternative strategies for caries prevention [15,16].

Initial dental caries detection occurs at the cavitation stage using simple visual and tactile inspection of the dental hard tissue. However, an accurate diagnosis of visible enamel decay requires further examination by optimal illumination and control of saliva under professional dental surgery settings. In addition, a cooperative child is fully required for proper examinations, and probing may cause iatrogenic damage to the enamel. Dental radiographs may be used to find clinically unidentified lesions in both the dentine and enamel. The use of Bitewing radiographs increases the detection rate of interproximal surface caries and reveals a considerable amount of carious surfaces and inadequate restorations, which at first may appear clinically sound or adequate. Over the last two decades, significant research has introduced several novel methods of early carious lesions detection methods. The most common diagnostic aids utilize laser fluorescence and electric caries meters. New, non-invasive techniques recently developed include Quantitative Light-induced Fluorescence, DIAGNOdent, Fibre-optic Transillumination, and Electrical Conductance [17]. While the results of these non-invasive techniques are generally promising, additional evidence validating its diagnostic accuracy to gold-standard methods are required to support their widespread adoption in the clinic. These early carious lesion detection methods will become highly invaluable if validated, given the importance of early-stage caries diagnosis. Early caries management interventions before cavitation and pulpal involvement will identify caries-active patients and those at increased risk of caries in the future.

Treatment of childhood caries can be conducted with different types of intervention depending on the progression of disease, the child’s age, level of cariogenic bacteria in biofilm, as well as the social, behavioral, dental, and medical history of the child (“risk of caries”). Risk factors are determined based upon: (1) an interview with the parent and (2) clinical assessment of the child. Caries management by risk assessment is an evidence-based caries management approach based on the caries balance/imbalance model between protective factors and pathological factors. Caries risk assessment requires a continuous monitoring process with reassessment factored in regularly given the non-static nature of individual caries status. The interval between oral health reviews must allow for flexibility based upon individual needs, assessment of disease levels, and risk of caries. Given this flexibility, it should be noted that the longest interval for oral health reviews for patients younger than 18 years are recommended to be no greater than 12 months [18].

Clinical treatments are dictated based upon certain criteria such as whether the carious lesion is cavitated or non-cavitated, and whether the lesion is active or arrested. Non-cavitated lesions can be arrested under the right conditions. Once a lesion has been cavitated, especially if dentin is involved, a dental restoration is usually required (“operative treatment”). The rule of thumb is that the earlier the treatment, the easier and less expensive the treatment will be compared to extensive decay that may develop if prolonged. Topical/local anesthetics and different degrees of sedation may be required in some cases to relieve pain and/or anxiety during treatment. A dental handpiece (“drill”) and other hand instrument tools are used to remove decayed dental hard tissue from a tooth. A laser and air-abrasion may be preferred by some dentists during caries removal.

Once caries are removed, the missing tooth structure requires a filling material to return the tooth to high function and aesthetics. Conventional restorative materials include dental amalgam, composite resin, and glass ionomer cement. When decay is too extensive, there may not be enough tooth structure remaining to allow for a restorative material to be placed within the tooth. Thus, a preformed crown may be required to place over the tooth. Traditionally, teeth are shaved down to make room for the stainless-steel crown, but crowns have also been used to seal caries into the tooth without cutting to prevent caries progression (Hall technique). In certain cases, endodontic therapy may be necessary for the restoration of a tooth. An extraction can also serve as a treatment option for dental caries in primary teeth, though it may cause space loss and consequent crowding in the permanent dentition.

Mechanical failures, such as fracture and marginal leakage, are common in dental restoration due to the physical and chemical mismatch between artificial materials and native dental hard tissue. In addition, food debris can easily accumulate on the surface of conventional dental materials, resulting in the formation of dental plaque-retentive sites. These plaque-retentive sites are the cause of recurrent caries and subsequent dislodge of the restoration. This process leads to a deleterious cycle of re-restoration that, with increasing size, ultimately shortens the tooth’s life expectancy. In response to these disadvantageous occurrences, there is growing research interest in synthesizing dental materials with similar physicochemical and mechanical properties as native enamel and which also exhibit superior antibacterial activities [19,20].

The process of dental caries formation is initiated by caries-specific pathogens that produce acids as a result of the decomposition of sugars. Overactive acid production interrupts tooth surface mineralization balance and induces the demineralization of dental hard tissues. Based on this progression, caries prevention requires a daily habitual process but can also be a repairable process by utilizing efficacious strategies that target the inhibition of caries pathogen biofilm formation, reduction of dental hard tissue demineralization, and promotion of remineralization. As an example of caries pathogen biofilm prevention, antimicrobial peptides, polypeptide substances produced by innate host defense mechanisms against broad-spectrum pathogenic microorganisms, combined with remineralizing agents, have been used for the management of dental caries [21,22]. The authors in this Special Issue will highlight other innovative methodologies to efficiently and effectively address caries prevention, with specific attention paid to childhood caries. We believe that the important findings from these studies will expand and build upon traditional health education approaches and models and improve participation and engagement in the oral health promotion in children. We hope that this in turn will also further highlight the online community awareness that currently supports improvements in public health outcomes.
